# Urine collection methods and dipstick testing in non-toilet-trained children

**DOI:** 10.1007/s00467-020-04742-w

**Published:** 2020-09-12

**Authors:** James Diviney, Mervyn S. Jaswon

**Affiliations:** 1 Department of Paediatrics, Whittington Hospital, London, UK; 2 Azrieli Faculty of Medicine, Bar-Ilan University, Safed, Israel

**Keywords:** UTI, Urinary tract infection, Urine sampling, Clean catch, In-out catheterisation, Suprapubic aspiration, Dipstick testing

## Abstract

Urinary tract infection is a commonly occurring paediatric infection associated with significant morbidity. Diagnosis is challenging as symptoms are non-specific and definitive diagnosis requires an uncontaminated urine sample to be obtained. Common techniques for sampling in non-toilet-trained children include clean catch, bag, pad, in-out catheterisation and suprapubic aspiration. The pros and cons of each method are examined in detail in this review. They differ significantly in frequency of use, contamination rates and acceptability to parents and clinicians. National guidance of which to use differs significantly internationally. No method is clearly superior. For non-invasive testing, clean catch sampling has a lower likelihood of contamination and can be made more efficient through stimulation of voiding in younger children. In invasive testing, suprapubic aspiration gives a lower likelihood of contamination, a high success rate and a low complication rate, but is considered painful and is not preferred by parents. Urine dipstick testing is validated for ruling in or out UTI provided that leucocyte esterase (LE) and nitrite testing are used in combination.

## Introduction

Urinary tract infection (UTI) is a common bacterial infection and, as such, a common presentation to paediatric health services. 5.9% of children presenting acutely to UK General Practitioners (family doctors) will have a UTI, rising to 7.3% if the population is restricted to those < 3 years old [1] and 7% of those < 2 years with urinary symptoms [2].

There are several common pathogens, with > 70% of cases due to *E. coli*, with c.10% involving other coliforms (including *Klebsiella* and *Enterobacter* species) and c. 5% *Proteus* species [3–5]. UTIs are not trivial infections, with detectable bacteraemia of the same organism in 10% of cases, rising to 17% in those < 1 month old [3]. 2.8–16% of individuals may develop kidney scarring following their first episode of UTI, with 8.4% of these developing hypertension, and a small proportion progressing to kidney failure [6–8]. Kidney scarring and damage can be prevented if UTI is treated in a timely manner, with delay leading to increasingly likely scarring [9, 10].

With this potentially avoidable morbidity, it is crucial to have the correct diagnosis. Clinical diagnosis is not straightforward and symptoms are non-specific, complicated by the fact that those who are most susceptible are least able to report symptoms. There is a reduction in incidence of UTI with advancing age, which is of significance given the higher prevalence of UTI in non-toilet-trained children. Meta-analysis of females with UTI shows a reduction in prevalence with advancing age, from 7.5% at 0–3 months, 5.7% at 3–6 months, 8.3% at 6–12 months and 2.1% in those > 12 months [2]. Overall, if there is significant suspicion, such that a urine sample is sent, then females are around three times more likely to have a UTI compared with males [4].

Given the high prevalence of UTI, diagnostic difficulty and associated severe morbidity with delayed treatment, it is important to use the optimal approach to obtain urine samples to confirm or exclude this important diagnosis. This is challenging in non-toilet-trained children.

Once obtained, urine needs to be tested and, although the definitive diagnosis is confirmed on urine culture, more immediate results are key for clinical decisions to be made, usually provided by dipstick testing or laboratory microscopy.

Publications were identified through searching Google Scholar, PubMed and Crossref with search terms relevant to paediatric urine collection. Inclusion for full review was based on abstract review. Reference lists of included publications were then hand searched for further relevant material.

In this review, we explore the relative merits of different methods of obtaining urine samples from non-toilet-trained children as well as reviewing the use of dipstick testing and microscopy in this patient group.

## Key guidance

Given the high prevalence and potential for morbidity from UTIs, there are several sources of key guidance published internationally, with several countries referencing the UK and US guidance.

UK NICE guidance [11] advises that clean catch urine sampling is the recommended methodology but that if this is unobtainable, then other non-invasive methods such as a urine collection pad (but not gauze, cotton wool or sanitary towels) should be used.

Where it “is not possible or practical” to collect urine non-invasively then catheter sampling or suprapubic aspiration (SPA) should be used. If SPA is used, then an ultrasound should be performed first to confirm urine in the bladder [11].

The American Academy of Paediatrics (AAP) guidance [12] recommends that for diagnosis of UTI if antibiotics are to be given, then the “specimen needs to be obtained through catheterization or SPA, because the diagnosis of UTI cannot be established reliably through culture of urine collected in a bag.”

If immediate antibiotics are not required, then they advise that there are two options: either “to obtain a urine specimen through catheterization or SPA for culture and urinalysis” or “to obtain a urine specimen through the most convenient means and to perform a urinalysis.” If the urinalysis results suggest a UTI (positive leucocyte esterase or nitrite test or microscopic analysis positive for leucocytes or bacteria), then a urine specimen should be obtained through catheterization or SPA and cultured. They do not recommend clean catch, pad or bag sampling.

Other international guidelines from Australia, Canada, the European Society for Paediatric Urology, Israel, Italy and New Zealand suggest the use of bag sampling in systemically well children, with an alternate method of collection required (clean catch, catheter or SPA) if dipstick from bag samples is suggestive of infection [13–18].

In unwell children, catheter or SPA sampling is recommended by Australian, Canadian, European, Italian, New Zealand, UK and US guidance [11–13, 15–17, 19].

There is a clear difference between international guidelines in how they advise urine is to be collected, with differing levels of technical challenge depending on the method employed. We review the benefits and disadvantages of each method of urine collection.

## Methods of collection

### Clean catch urine

Clean catch urine (CCU) samples are obtained by holding a sterile container beneath the urethra with the nappy off, until a void begins, with care taken to avoid any skin contact with the specimen container. Voiding can be stimulated as detailed in a later section of this review.

Contamination rate is a key differentiator determining collection approaches as it is estimated that 30% of contaminated samples are masking true UTIs [20]. Reported contamination rates differ significantly by approach and vary widely between sources. Published rates are collated in Table 1 for ease of comparison.

**Table 1:** Comparison of contamination rates by collection method

Method	Contamination rates reported
Clean catch	4.5% [21] 14.7% [22], 26% [23], 27% [24], 16–38% [25]
Bag	18% [26], 26.6% [22], 36.8% [27], 43.9%[20], 45% [28],46% [23], 62.8% [29], 68% [24] and 88% [12]
Pad	9.1% [21], 16% [26], 29% [22], 65% [24] and 80% [22]
Catheterisation	8% [30], 9–23% [25], 9.1% [29], 12% [23], 14.3% [20] and 28.6% [31].
Suprapubic aspiration (SPA)	0–7% [25]*,* 1% [23] and 9.1% [20]

NICE recommends clean catch sampling and SPA as the most diagnostically accurate sampling methods. As shown in Table 1, there are several sources demonstrating catheterisation as having a lower contamination rate than that reported for clean catch sampling, but in direct comparison clean catch compares well to catheter sampling with a contamination rate of 5%, compared with 8% for catheter samples [32]. In sources without direct comparison, perineal cleaning can also reduce the contamination rate comparable with that published for catheter sampling, as discussed below.

Contamination rates seem to differ by gender with 10.5% of male samples and 16.4% of female samples being contaminated [25]. Other sources also find contamination is significantly higher in females and in those aged 0–3 months and over 12 years or if the urine sample is collected at home [4, 33].

SPA is generally used as the comparator for contamination rates as it involves the least exposure to non-sterile sites such as the distal urethra. When clean catch sampling is compared directly to SPA, sensitivity ranges from 75 to 100% and specificity ranges from 57 to 100% [34]. A separate examination of clean catch sampling compared with SPA as a gold standard reported a sensitivity of 88.9% and a specificity of 95.0%, giving false-positive rates of 5% and false-negative rates of 12% compared with SPA [35].

By its nature, clean catch sampling is time consuming, with a median time to sample of 30.5 min and an interquartile range of 11–66 min [28]. Seventy-five percent of CCUs are obtained in 1 h and 25% of these within 7 min [36]. It is also relatively easy to “miss” collecting a sample from a voiding event, occurring in around 16% of attempts [28]. A combination of these factors may contribute to failed collection with families abandoning attempts in 20% of cases [28]. If a sample is not achieved by 1 h, then additional time taken is unlikely to lead to a sampling success [28], a useful parameter to consider in busy departments where patients may be kept from going home while awaiting a sample.

Clean catch samples, as expected, are easier to obtain in older children. The DUTY (Diagnosis of UTI in Young Children) study found a significant difference in method of collection by age in UK primary care cases: of 2884 children aged < 3 years, 26.3% of samples were collected using clean catch vs. 96.7% of children aged 3–5 years [37].

#### Perineal cleaning

Practice differs between centres as to if and how the perineum should be cleaned before a clean catch sample is collected, with some centres using saline, soap or chlorhexidine.

Interventional studies have shown that a staff education package highlighting the importance of cleaning did not change contamination rates in an emergency department but as monthly clinical activity increased, so did the likelihood of contamination, suggesting it is an avoidable occurrence [33]. A randomised trial of cleaning with soap vs. not cleaning in 350 patients found significantly lower contamination rates in the cleaning group (7.8%) vs. the non-cleaning group (23.9%) [38]. In a different study, perineal cleaning reportedly improved contamination rates with a contamination rate of 5% after cleaning, lower than most reported rates [30].

A study focussed on targeted cleaning in uncircumcised boys found no change in contamination rates [39]. However, a systematic review provided overall support for perineal cleaning and collection of a mid-stream rather than initial stream sampling [40].

### Bag sampling

Bag sampling involves attaching a sterile plastic bag to the perineum, usually with adhesive around the bag opening, such that voided urine falls within the bag.

While actively recommended against by the AAP, bag sampling remains a highly utilised method of sampling, especially in the community. It is the preferred method of collection in Europe: of 1129 paediatricians surveyed, 53% selected a bag as their first choice for infants < 3 months and 59% for children 4–36 months of age [41], whereas in the USA, 25% of samples for 3066 infants were collected by bag, 70% by catheter, 3% by SPA and 2% by clean catch [3]. The ease of bag sampling lends itself well to a community setting.

There is a clinician concern that the adhesion of the bag to sensitive skin causes moderate to severe pain when assessed on visual pain scales [42]. A study of French doctors reported bag removal as “equally to more painful” than catheter removal for females and “equally to less painful” for males [43]. Reports on the practicalities of sampling note that the bag pulls away from the skin when full and heavy, leading to sample loss [44].

Contamination rates in Table 1 are higher than for clean catch sampling but rates are not correlated with the time taken to obtain the sample [28]. The AAP guideline states that bag cultures have “an unacceptably high false-positive rate and are valid only when they yield negative results”, stating that the rate of false positives range from 88 to 99% of tests [12].

In centres where suprapubic aspiration is rarely performed, catheter samples may be used as a reference standard. When directly compared with catheter samples in the same children, 7.5% of bag samples were false positives and 29% false negatives [45], compared with a false-positive rate of 18% and false-negative rate of 24% in another study [46].

### Pad sampling

Pad sampling involves the insertion of an absorbent material into the child’s nappy from which urine is later aspirated after voiding. There are custom designed pads for this purpose, such as the Newcastle urine collection pad (UCP).

The main advantages of the pad collection are that the process is passive and requires less parental effort with less disruption to the child. There is decreased likelihood of missing a sample compared with clean catch collection, with overall sampling success of 96% [22]. It is the preferred collection method of parents [26].

In direct comparison the time taken to obtain a pad sample is shorter than for clean catch, with a median of 30 vs. 107.5min, however this duration is comparable to the 30.5 min median collection time for clean catch sampling reported by other publications [21, 28]. One source reports a longer median collection time for pad sampling of 45 min [47].

UCPs trap and retain a substantial amount of cellular material, reducing cell counts on microscopy but dipstick testing for blood and leucocyte esterase remains reliable [48]. Several sources have shown microscopy of pad samples gives a lower white cell count [49–51].

Some centres use cotton wool in place of specific UCPs. This is problematic as cotton wool balls have antibacterial properties. In one study, *E. coli* was shown to be unaffected by cotton wool, but colony counts of *Enterococcus faecalis* fell by up to 75% after 30 min of contact, with complete elimination of the organism after 2 h [50]. Cotton wool also impacts on viral studies: absorption of CMV onto cotton and the inhibitory effect of formalin reduce CMV counts on rapid culture but do not alter PCR results [52].

Contamination is the main concern in the use of UCPs, with prolonged contact between the pad and perineum thought to increase the likelihood of contamination with skin or gut flora. Measures to reduce this risk have been trialled including use of moisture-sensitive alarms, which do not reduce contamination [53] and changing the pad every 30 min. In a randomised controlled trial, changing the pad every 30 min gave a contamination rate of 3%, compared with 29% when a single pad was left in place [47].

Likelihood of sample contamination has been found to be significantly increased in pads when compared directly with clean catch samples, with pads contaminated in 12.2–26.3% of cases vs. 1.8–6.4% for clean catch [37]. In the DUTY study of 2740 clean catch vs. 2277 nappy pad samples, the risk ratio of contamination was 6.66 for pads [4]. The probability of sample contamination was not increased by delay in the time taken for samples to arrive at laboratories nor the presence of a nappy rash. However, it was increased in female patients or with parents collecting samples at home by either method [4]. In the same study, the contamination rate with coagulase-negative *Staphylococcus* in pads was 1.8 times higher than for clean catch urine but contamination with faecal organisms (*E. coli* and *Enterococcus*) was not significantly increased [4]. Contamination with *E. coli* is especially problematic as it is both a common contaminant and the most common UTI pathogen.

The prevalence of UTIs diagnosed was lower for nappy pad (1.3%) than for clean catch (2.3%) samples, suggesting that UTIs are missed in nappy pad samples because of contamination [4]. The presence of squamous epithelial cells on microscopy helps predict contamination in clean catch urine (indicating passage of urine over the skin) but not in nappy pad samples (where there is sustained skin contact) and, as such, the presence or absence of squamous cells should be ignored in nappy pad sampling [4].

#### Urinalysis from pad samples

While paediatric practice commonly requires sterile collection of urine for potential bacterial culture, there are other reasons that urine may be collected. If sterility is not required, then pad collection can be advantageous.

Importantly, pads have been shown not to clinically impact levels of nitrites, glucose, ketones, urea, electrolytes, creatinine, osmolality, calcium, phosphate, magnesium, urate, oxalate, pH, toxicology for drugs of abuse, catecholamines, amino acids, organic acids and glycosaminoglycans [48, 54]. Pads show good concordance to other methods of sampling for diagnostic urinary metabolites in patients with metabolic disorders including phenylketonuria, cystinuria, mucopolysaccharidoses II and III, organic acid disorders and altered urine simulating urea cycle disorders [54].

However, it is important to note that some nappy materials and cotton wool balls selectively absorb creatinine [55] and protein [56]. Albumin binding to pads is very variable and can be up to 10–40% of sample content, with retinal binding protein also adhering to pads within 15 min of contact [48, 56]. Protein retention increases the longer the urine is left in a pad, reaching 20–30% after 90 min [48].

### In-out catheterisation

In-out catheterisation describes the temporary insertion of a urinary catheter to obtain a sample, with the catheter then being removed. Theoretical benefits are that this avoids some contamination from colonising bacteria in the distal urethra compared with voided samples and may be less likely to lead to complications than suprapubic aspiration. Best practice is to discard the initial few drops of urine which are thought to have a higher likelihood of contamination from urethral bacteria, but this was not described in most of the sources reviewed. A randomised comparison of early sampling or late sampling found significantly higher sample contamination if the first drops of urine collected were cultured [57].

Test performance for cultures from in-out catheterisation is better than for non-invasive methods, with urine obtained for culture by catheter having a sensitivity of 95% and a specificity of 99%, compared with urine obtained through SPA [58–60].

One study examined the clinical impact of a contamination rate of 9.1% in catheter sampling. They found that the odds ratios of adverse events were 4.9 for unnecessary recall for further testing, 4.8 for unnecessary treatment, 15.6 for unnecessary prolonged treatment, 4.1 for unnecessary radiologic investigation and 12.4 for unnecessary hospital admission [29]. When SPA is performed to confirm organisms cultured from a catheter sample, it has identified a false-positive rate of 71% from catheter samples [61].

Catheterisation is a less variable procedure than suprapubic aspiration, with authors reporting a 92.3% [45] to 100% success rate [62]. Use in a neonatal population reports an 81.2% [63] to 83.3% success rate [64].

Significant complications are rarely described, with transient microscopic haematuria in 17% [65]. One study found that previous catheterisation was a risk factor for septicaemia in neonates [66].

Practitioner perception is that catheterisation is painful with most European centres surveyed using some form of analgesia [41]. However, intermittent self-catheterisation is well tolerated by children with intact sensation suggesting that it is not inherently painful [67, 68]. Staff confidence is reported to be low with half of junior doctors and nurses surveyed not having had training in how to catheterise children [69].

### Suprapubic sampling

Suprapubic aspiration is undertaken through the insertion of a 22G needle through the anterior abdominal wall and into the bladder, usually aided by ultrasound guidance, with urine aspirated into a syringe.

Suprapubic sampling is often described as the gold standard for urine sampling due to the theoretical minimal likelihood of contamination [44], avoiding bacteria which colonise the distal urethra as normal flora [70].

However, SPA is also considered the most invasive and painful method by practitioners and parents [61]. In contemporaneous scoring by parents and nurses in infants < 60 days old, it was rated as more painful than catheterisation [64].

The success rate varies by whether ultrasound guidance is used. Reported success rates in unguided attempts were 46% [62] to 64% [70]. Under guidance success improves from 79% [71] to 90% [70]. In direct comparison, there is reported improvement with guidance from 36 to 100% [72], 60 to 96% [73], 62 to 93% [35] and 52 to 79% [71]. As in many procedures, experience is beneficial, with improved success rates noted as the study progressed [70].

Patient selection and preparation are also important. There was no difference in success noted with or without guidance in one study if the patients were prehydrated and had identifiable dullness on suprapubic percussion: first attempt success was 60% in both groups, rising to 87% guided vs. 80% unguided if 3 attempts were made [74]. In one study employing bladder measurements, there were no successful attempts if the antero-posterior diameter of the bladder on ultrasound was < 2 cm [75].

In neonatal patients, an unguided approach gave 64.7% success on the first tap [63]. Guidance may be of less benefit in the neonatal population with a success rate of 75% guided vs. 74% unguided in those under 28 days old [70].

Complications are rare, occurring with a rate of only 0.22% of 4985 SPAs performed in one study [76]. However, other studies have noted aspiration of gut lumen contents in 1 in 140 aspirations [70]. Microscopic haematuria has been seen for 24 h post aspiration in 3.6% of patients [70].

Any growth on urine cultured from SPA may be taken as significant given the sterile nature of the procedure [77, 78], but some centres still apply a culture threshold, but at a lower level than for sampling by other routes. The Italian Society for Paediatric Nephrology recommends a threshold of 1 × 10^4^ cfu/ml from catheter or SPA samples, 5 × 10^4^ cfu/ml from clean catch and 1 × 10^5^ cfu/ml from bag samples [17]. The American Academy of Paediatrics suggests 5 × 10^4^ cfu/ml as the threshold for UTI in children (regardless of collection method) under the age of 2 years [12], used in combination with pyuria to determine if the organism is a true culture, or a contaminant if pyuria is absent. Laboratory standards for the UK advise that 10^3^ cfu/mL of a single species “may be diagnostic of UTI” and 10^4^–10^5^ cfu/mL “is indicative of UTI in a carefully taken specimen” [78].

### Stimulation of voiding

Non-invasive testing relies on spontaneous voiding by the child. Stimulation of voiding could help achieve samples in a timely manner, allowing less time for contamination to occur.

In neonates, where central inhibition of spinal reflex arcs is less developed, voiding can be stimulated by holding the baby upright under both armpits with the legs dangling, tapping the abdomen suprapubically at 100 taps/min for 30 s, followed by circular lumbosacral massage for 30 s, alternating for up to 5 min. This has been shown to promote voiding, especially if performed 30 min post feeding [79]. Using this technique in those < 7 days old led to voiding within 5 min in 90% of infants [80].

In a population with mean age of 6–7 days, this technique gave a median collection time of 45 s with success in 86.3% [79]. In direct comparison of infants < 10 days old, 78% voided in 5 min with stimulation vs. 33% of controls without stimulation [31].

The effectiveness of the technique declines with age. In an older population of those < 6 months, 49% voided within 5 min of stimulation with a median collection time of 45 s [81]. A different study found that in a population with a median age of 10 months, the success rate was 27%, with most successful attempts occurring within 2 min [82]. They also found that likelihood of success decreased as the weight of the child increased [82].

Suprapubic and lumbosacral stimulation is not the only method of voiding stimulation that has been investigated. The “Quick-Wee” technique has been reported where chilled (2.8°), saline-soaked gauze is rubbed on the suprapubic area. In children aged 1–12 months, if it was stimulated for up to a 5-min period 31% of children voided vs. 12% of unstimulated control patients [83]. Fewer possible collection opportunities were missed: only 2 in the stimulation group vs. 5 in the control group [83]. There was no reported difference in the contamination rate, being around 27%, which is similar to other clean catch sample studies [83]. Use of ultrasound to determine bladder fullness before attempting stimulation did not lead to improved sample collection within 5 min [84].

## Dipstick testing for UTI

Following successful sampling, the urine can be tested using point of care dipstick analysis and/or sent to the laboratory for microscopy and culture. The advantage of point of care dipstick testing is that this provides information to guide clinical decision-making immediately. The panel of routine tests for infection includes leucocyte esterase and nitrite.

### Leucocyte esterase

Leucocyte esterase (LE) is an enzyme produced by white cells and is found in urine where white cells have been active (pyuria), such as when there is active infection.

Use of LE testing should be taken in the context of the clinical picture, with a high rate of false negatives in neutropenic patients [85] and in younger children whose frequent voiding reduces LE accumulation in stored urine [86]. As LE is not exclusively raised in children with UTI, the presence of pyuria does not entirely exclude that an organism cultured is a contaminant [4].

The sensitivity and specificity of LE for UTI varies by sample collection method. If a positive result for LE is taken as “+” or above, then sensitivity/specificity is reported as 86%/80% from clean catch [30], 86–88%/94–100% from catheter sampling [30, 66] and 76%/84% [66] from bag sampling with an overall sensitivity/specificity of 84%/91% [66]. A different study found no change in sensitivity/specificity of LE after urine was left for 2 h in contact with incontinence pads [87].

A meta-analysis examining LE reported significant heterogeneity between the studies with sensitivity ranging from 37.5% (specificity 96.4%) to 100% (specificity 92%) and specificity ranging from 69.3% (sensitivity 93.5%) to 97.8% (sensitivity 70%). The pooled positive likelihood ratio (LR+) for UTI was 5.5 and the pooled negative likelihood ratio (LR−) was 0.26 [34].

The extent of positivity (trace to +++) is also a useful parameter to assess. The DUTY study of over 7163 patients found that from samples collected from pads, the odds ratios of UTI are outlined in Table 2.

**Table 2:** Odds ratio of urinary tract infection (UTI) by extent of leucocyte esterase (LE) positivity for pad and clean catch sampling

Extent of positivity of LE	Odds ratio of UTI from pad sampling [37]	Odds ratio of UTI from clean catch sampling [4]
Trace	0.87 (non-significant)	5.4 (significant)
+	2.06 (non-significant)	2.47 (non-significant)
++	1.63 (non-significant)	19.61 (significant)
+++	3.27 (significant)	66.6 (significant)

Asymptomatic bacteriuria as well as contamination should be considered in children with a positive culture result but a negative LE result [12]. This is an uncommon phenomenon, with a meta-analysis including 49,806 children showing asymptomatic bacteriuria in 0.18% of boys and 0.38% of girls [88].

### Nitrite

Nitrite is produced from dietary nitrate in the bladder through metabolism by bacteria. It compares with LE in having lower sensitivity but higher specificity for UTI testing [12]. Nitrite is not associated with all organisms as it is not produced by *Pseudomonas* species or gram-positive organisms such as *Enterococcus* [85].

Nitrite testing is also impacted by age, as the more frequent voiding of younger children allows less time for nitrite to accumulate prior to sampling [86]. Original studies quote a dwell time of 4 h in the bladder to convert dietary nitrates to nitrites [89]. However, one paper goes against this trend, finding an increased nitrite positivity in those < 3 years with UTI 12.9% vs. 2.2% in those over 3 years [90]. Children may also have diets too poor in fruit and vegetables to provide sufficient urinary nitrate for conversion [91] or the nitrate ions present may degrade due to the lower sodium concentrations, more acidic pH or presence of urobilinogen more commonly seen in younger children [92].

As it is a product of bacterial metabolism, nitrite continues to be produced in urine samples after voiding, potentially changing a false-negative to a true-positive result over time. A study in adults found 0.5% of samples went from negative to positive after 2 h in an incontinence pad [93].

A meta-analysis covering 95,703 children found the sensitivity/specificity of nitrite testing to be 49%/98% [94], a different meta-analysis found wider ranges of sensitivity, from 16.2 to 88.1% with specificity ranging from 75.6 to 100%; all but two estimates of specificity were above 90% [34]. Smaller individual studies report sensitivity/specificity of 24–50%/98–100% [30, 66, 85].

A different meta-analysis found the pooled LR+ for UTI from positive nitrite testing to be 15.9 with a LR− of 0.51 [95]. The DUTY study found that from samples collected from pads, the odds ratios of UTI for nitrite positivity was 3.16 [37] compared with 38.4 for clean catch urine samples [4].

### Combination testing

Taking these parameters in combination can improve the sensitivity and specificity compared with testing each in isolation. A dipstick can be taken as indicating UTI if LE *or* nitrite is positive or alternately can only be considered positive if both LE *and* nitrite results are positive. Table 3 collates information from a variety of meta-analyses. While the LR+ is repeatedly reported as significantly better for the “and” comparison, the LR− is not significantly different between “and” and “or” in some of the meta-analyses.

**Table 3:** Likelihood ratio of urinary tract infection (UTI) depending on interpretation of leucocyte esterase (LE) and nitrite results

Test condition	Positive likelihood ratio for UTI	Negative likelihood ratio for UTI	Sensitivity/specificity for UTI
LE *or* nitrite positivity/negativity	6.1 (meta-analysis of 15 studies) [95]	0.20 (meta-analysis of 15 studies) [95]	69.4–100%/69.2–97.8% (meta-analysis of 15 studies) [34] 88%/79% (meta-analysis of 95,703 children) [94]
LE *and* nitrite positivity/negativity	28.2 (meta-analysis of 9 studies) [95] 22.8 (study of 5017 samples) [4]	0.37 (meta-analysis of 9 studies) [95] 0.22 (study of 5017 samples) [4]	30–89.2%/> 90% (meta-analysis of 15 studies) [95] 92.5%/39.4% [96] 82.5%/81.3% [5]

In the DUTY study, dipstick negative for both LE and nitrite had a pooled LR− of 0.22, suggesting that a dipstick negative for both nitrite and LE may be useful in ruling out a diagnosis of UTI [4]. Likewise, the pooled LR+ was 22.8, suggesting that a dipstick positive for both LE and nitrite may be useful for ruling in a UTI [4]. Individually however, the sensitivity/specificity of LE and nitrite was too variable to allow them to be of any use in exclusion or inclusion of UTI [4].

The method of sample collection also influences combined sensitivity/specificity, with bag sample dipstick testing shown to be more sensitive than catheter testing: 85% vs. 71% [97] but less specific. 62% vs. 97%, respectively [97]. This likely represents the trade-off of lower specificity giving higher sensitivity, with fortuitous capture of UTI in some contaminated bag samples. There was no significant difference between clean catch and catheter sampling in the performance of combined LE/nitrite testing in those under 90 days old with sensitivity/specificity of 86%/80% for clean catch and 91%/95% for catheter samples [30].

Overall, a combination of positive results for LE and nitrite is most accurate for ruling in disease, and a negative test for both nitrite and LE is the most accurate for ruling out disease [34]. However, the DUTY study of over 6390 samples identified that dipstick testing led to 3.3% of children without UTI being incorrectly treated for UTI vs. a rate of 2.3% for lab microscopy [98].

A meta-analysis by Whiting et al. showed that dipstick negative for LE *and* nitrite or microscopy negative for pyuria and bacteriuria can be reasonably used to exclude UTI in urine obtained from clean catch, bag or pad samples [95].

### Age-related test performance

The sensitivity and specificity of combined analysis changes with increasing age, with test performance improving. This, combined with the greater prevalence of UTI and likelihood of bacteraemia in young infants, impacts on how dipstick testing should be used.

NICE recommends that urine dipstick testing is not used in patients < 3 months old due to worse diagnostic performance in younger age groups. For 3 months–3 years of age, they recommend that dipstick testing can be used to rule in UTI and rule it out in otherwise well children, without prior recurrent UTI. In those over 3 years, dipstick testing can be used to rule in and out UTI with positive nitrite being more indicative of UTI and necessitating treatment immediately, whereas positive LE can await culture results [11].

This reflects work by Sharief et al. and Shaw et al. who showed poor performance for LE and nitrite in those younger than 2 years [99, 100]. In meta-analysis, these two sources show that using LE and nitrite give a LR+ of 7.74 for children < 2 years and 28.79 for children > 2 years. Likewise, the LR− is 0.32 in the younger group and 0.19 in the older group [11]. A different meta-analysis also incorporating these studies and comparing dipstick performance to microscopy with stratification for age found that the highest LR+ was for dipstick testing in children over 2 years old (dipstick 27.1 vs. microscopy 1.69) and for microscopy in those under 2 years (dipstick 6.24 vs. microscopy 15.6). To rule out infection, the lowest LR− was for microscopy in all age groups (< 2 years = 0.27, > 2 years = 0.04) but in those under 2 years, it was comparable with dipstick testing (LR− 0.31 for dipstick vs. 0.27 for microscopy) [101].

A meta-analysis of eighteen papers by Coulthard examining age-related performance of nitrite testing [102] found that every study showed lower mean sensitivity of nitrite in UTI for children < 2 years old, with significantly poorer pooled sensitivity values of 0.38 vs. 0.72 for those >2 years old. The included RCTs showed an even more marked difference when analysed separately (0.23 vs. 0.81). There was no clinically significant impact of age on nitrite specificity. A separate meta-analysis found no difference in the sensitivity of combined dipstick testing in those above and below 2 years of age, noting significant heterogeneity between the sources within each age group [103].

In additional work, sensitivity/specificity in patients aged 0–2 years was 87.5%/39.7% vs. 100%/39.7% in those 2–10 years old, with a negative predictive value of 100% [96]. Given the 100% sensitivity, this suggests UTI can be adequately excluded in children 2–10 years with negative combined testing.

A further study reports that combined nitrite and LE urinalysis shows sensitivity increasing as patient age increases, from 75% in less than 2 months to 83.5% in those over 2 years, with specificity decreasing from 90.3 to 75.1%, respectively [5]. Likewise, a separate paper showed an increase in sensitivity from 69% in infants < 90 days old to 88% in those older than 90 days [97].

### Blood

Haematuria is strongly associated with UTI, independent of LE and nitrite [4, 104]. However, haematuria cannot be used to rule in or rule out UTI, with a meta-analysis reporting an estimated sensitivity of 53.3% and specificity of around 85% [34].

Some bacterial peroxidases may cause false-positive results for blood where no haemoglobin is present in the urine [105]. Dipstick results positive for blood are also associated with higher likelihood of the sample growing contaminants when cultured [4, 105].

## Microscopy

Microscopy of a urine sample in the context of infection can provide information about the presence of bacteriuria and pyuria. In isolation, bacteriuria is superior to pyuria in ruling in and out UTI (LR+/LR− of 14.7/0.19 vs. 5.9/0.27, respectively) [95]. However, the best test performance comes from combining pyuria and bacteriuria with both being present giving a LR+ of UTI of 37.0 and the absence of both giving a LR− of 0.21 [95]. The meta-analysis of 39 studies by Whiting et al. noted significant variance in the quality of reporting of pyuria between studies and centres [95].

Meta-analysis of attempts to combine dipstick and microscopy testing showed such high variance in the positive and negative likelihood ratios that no overall conclusion can be drawn [95].

In a different meta-analysis, microscopy for bacteriuria post gram stain was found to be the most accurate of any dipstick or microscopy parameter with sensitivity/specificity of 91%/96%, including comparison with LE and nitrite in combination [94]. Even this as the best parameter still has a high false-negative rate of 9% [94]. The same analysis highlighted that LE dipstick performance was identical to microscopy for pyuria, while being technically much easier and faster. However, a different meta-analysis found that the LR− of absence of pyuria and bacteriuria was 0.11, vs. a LR− for LE and nitrite of 0.37, suggesting microscopy is superior to dipstick in ruling out UTI [34].

## Costs

The DUTY study extensively examined the economic and clinical costs of urine testing in a primary care setting. They directly compared laboratory testing and dipstick testing, finding that for children deemed to have an intermediate risk of UTI, dipstick testing was less cost effective than laboratory-based testing, due to the greater number of children incorrectly started on antibiotics (3.3% vs. 2.3%) and the additional time and cost of dipstick testing. However, the clinical benefits and quality-adjusted life days were very similar between each strategy [98].

The greatest determinant of the economic cost of testing a urine sample in secondary care is the time spent occupying a hospital bed rather than the equipment used [106]. The costs calculated in 2020 to obtain an uncontaminated sample are as follows: catheterisation £49.39, SPA £51.84, stimulated void £52.25, clean catch £64.82 and urine bag £112.28 [106]. This reflects a lower likelihood of contamination resulting in less repeated testing and more rapid sample collection requiring less time in the hospital.

As with any cost analysis, the expense of over-investigation (estimated 2011 cost of an ultrasound scan being £50 and micturating cystourethrogram (MCUG) being £137) is offset against reduction in long-term clinical and economic costs of under-investigation (dialysis costing £21,655 a year) [98]. The DUTY study found that while more sensitive urine sampling strategies identify a higher proportion of vesicoureteric reflux (VUR) initially, the benefits are not significant at a population level given the moderate effectiveness of treatment for (VUR) and the low likelihood that kidney scarring progresses to kidney failure [98]. The best approach was to carefully select the children for whom a sample would be sent, with the study providing a model to support this decision, which performed superiorly compared with clinical judgement and gave a net benefit of £0.42 per patient with marginally better short- and long-term outcomes [98].

## Conclusion

Diagnosis of UTI requires an uncontaminated urine sample for microscopy and culture. This is challenging in non-toilet-trained children and several approaches exist. No methodology is perfect but we recommend clean catch sampling, in combination with techniques to stimulate voiding and perineal cleaning, as this canprovide rapid, non-invasive urine samples that can be sent for microscopy and culture. The contamination rate of clean catch sampling compares well to invasive catheterisation and outperforms pad and bag sampling.

In a primary care setting, clean catch sampling as guided by parent-reported symptoms, physical examination and dipstick testing is a cost-effective and appropriate approach for clinical decision-making.

In secondary care, the major cost of diagnosing UTI is the time spent in hospital obtaining a sample. There is potential for this period to be reduced through the increased use of voiding stimulation techniques.

When invasive collection is required in an unwell child or when non-invasive methods are unsuccessful, catheter samples show higher levels of contamination than suprapubic aspiration but are better tolerated by patients and families.

Once a sample is obtained, urine dipstick testing can be used in children over 3 months old and positive or negative results for both leucocyte esterase and nitrite on testing should be used to rule in and out a UTI, respectively. A negative urinary nitrite alone should not be used to exclude UTI in children less than 2 years old while a positive nitrite in any child is very strongly supportive of UTI.

Microscopy for pyuria and bacteriuria performs better than dipstick in ruling out UTI in children overall, but is comparable with dipstick testing in those under 2 years of age, performing sufficiently well to exclude UTI however urine has been collected. This is significant in that bag, pad or clean catch samples can be dipped and children with negative results excluded from further investigation, as supported by a key meta-analysis by Whiting et al. Although microscopy performs well, it is not offered by all laboratories nor generally available at the point of care.

However, it should be noted that there is significant variation in the results of the sources referenced in this review which examine the performance of all aspects of urine testing. We have made our recommendations on the best evidence available. There is a need for more tightly controlled and highly powered research, such as the DUTY study, examining the relative performance of collection methods and urine analysis to help address this.

The main message to take away from this review is that clinicians practicing within paediatrics should balance the risk of sample contamination, patient age, ease of sampling and acceptability to patients in selecting their collection method. Although pad and bag samples can be dipstick tested to exclude children from further investigations, sending possibly contaminated urine samples results in more invasive testing for children without UTI. We therefore recommend clean catch sampling following perineal cleaning and with the use of voiding stimulation techniques as representing an evidence backed, diagnostically accurate and acceptable choice in most cases.
