# Correction to: Urine collection methods and dipstick testing in non-toilet-trained children

**DOI:** 10.1007/s00467-020-04824-9

**Published:** 2020-11-23

**Authors:** James Diviney, Mervyn S. Jaswon

**Affiliations:** 1 Department of Paediatrics, Whittington Hospital, London, UK; 2 Azrieli Faculty of Medicine, Bar-Ilan University, Safed, Israel

**Correction to: Pediatr Nephrol**

10.1007/s00467-020-04742-w

The original version of the letter unfortunately contained a mistake. The catheterisation cost cited in the paper should be £49.39 instead of £25.98. The corrected paragraph is shown below. The original article was corrected.

“The greatest determinant of the economic cost of testing a urine sample in secondary care is the time spent occupying a hospital bed rather than the equipment used [106]. The costs calculated in 2020 to obtain an uncontaminated sample are as follows: catheterisation £49.39, SPA £51.84, stimulated void £52.25, clean catch £64.82 and urine bag £112.28 [106]. This reflects a lower likelihood of contamination resulting in less repeated testing and more rapid sample collection requiring less time in the hospital.”

